# Partitioning of gene expression among zebrafish photoreceptor subtypes

**DOI:** 10.1038/s41598-021-96837-z

**Published:** 2021-08-30

**Authors:** Yohey Ogawa, Joseph C. Corbo

**Affiliations:** Department of Pathology and Immunology, Washington University School of Medicine, 660 South Euclid Avenue, St. Louis, MO 63110-1093 USA

**Keywords:** Colour vision, Retina, Transcription, Molecular evolution

## Abstract

Vertebrate photoreceptors are categorized into two broad classes, rods and cones, responsible for dim- and bright-light vision, respectively. While many molecular features that distinguish rods and cones are known, gene expression differences among cone subtypes remain poorly understood. Teleost fishes are renowned for the diversity of their photoreceptor systems. Here, we used single-cell RNA-seq to profile adult photoreceptors in zebrafish, a teleost. We found that in addition to the four canonical zebrafish cone types, there exist subpopulations of green and red cones (previously shown to be located in the ventral retina) that express red-shifted opsin paralogs (*opn1mw4* or *opn1lw1*) as well as a unique combination of cone phototransduction genes. Furthermore, the expression of many paralogous phototransduction genes is partitioned among cone subtypes, analogous to the partitioning of the phototransduction paralogs between rods and cones seen across vertebrates. The partitioned cone-gene pairs arose via the teleost-specific whole-genome duplication or later clade-specific gene duplications. We also discovered that cone subtypes express distinct transcriptional regulators, including many factors not previously implicated in photoreceptor development or differentiation. Overall, our work suggests that partitioning of paralogous gene expression via the action of differentially expressed transcriptional regulators enables diversification of cone subtypes in teleosts.

## Introduction

**Figure 1:**
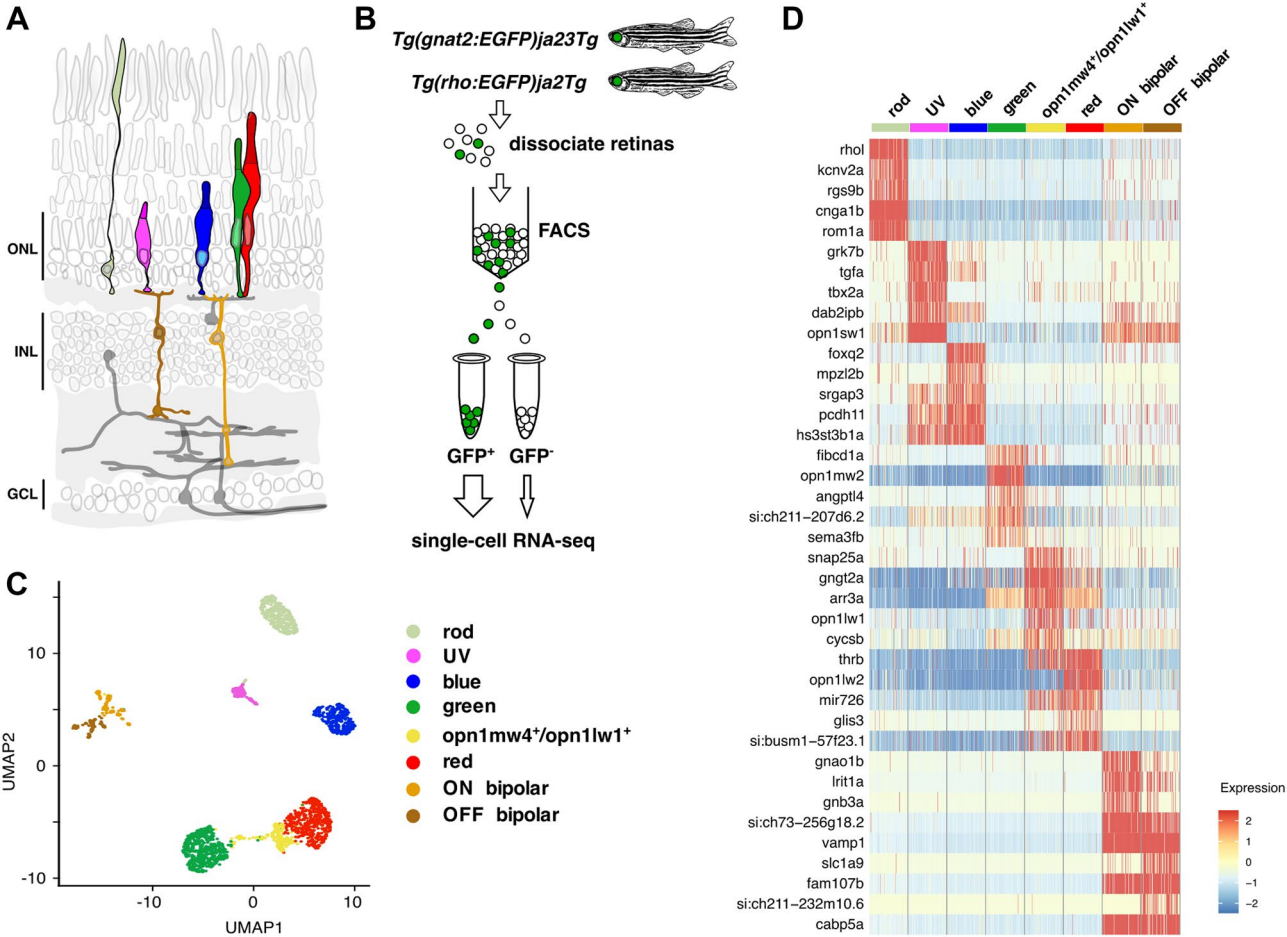
Transcriptome profiles of adult zebrafish photoreceptor subtypes. (**A**) Schematic representation of the major cell classes in the zebrafish retina based on a prior design^7^. Photoreceptor cell types and ON and OFF bipolar cells are highlighted in color, whereas other retinal cell types are in grey. The ON bipolar cell cluster in our single cell data expresses genes specific to both rod ON bipolar cells (*prkcaa*) and cone ON bipolar cells (*gnao1b, gnb3a, trpm1a, rgs11,* and *isl1*). See also Fig. S1. ONL outer nuclear layer, INL inner nuclear layer, GCL ganglion cell layer. (**B**) Isolation of rod and cone cells from transgenic adult zebrafish expressing green fluorescent protein (GFP). GFP-positive cells were collected from each line. A small percentage of GFP-negative cells was also included in the analysis. (**C**) Automatic clustering of single-cell expression profiles reveals six distinct photoreceptor populations. The plot shows a two-dimensional representation (UMAP) of global gene expression relationships among 2186 cells. (**D**) Heatmap showing top five differentially enriched genes for each cell population (rows). Columns correspond to single cells grouped by cell cluster. Each cell cluster is colored as in panel (**C**). Values are row-wise Z-scored gene-expression values*.* See also Fig. S1A. Full list of differentially enriched genes is provided in Supplementary Data S1.

In vertebrates, photoreceptor cells are categorized into two classes, rods and cones, which together are able to respond to a broad range of light intensities from dim starlight to bright sunshine^1–3^. Rods are primarily responsible for dim-light vision at night, whereas cones mediate bright-light vision and color discrimination^4^. Visual pigments, consisting of an opsin and a covalently bound chromophore, are the light-sensitive molecules of photoreceptors^5,6^. Absorption of a photon by a visual pigment activates the phototransduction cascade, which induces photoreceptor hyperpolarization and synaptic transmission to second-order neurons^7^ (Fig. 1A). Phototransduction pathways in rods and cones are composed of distinct opsins and signal transduction components^8^. Rod- and cone-specific phototransduction genes arose before or during two rounds of whole-genome duplication, which occurred in a chordate ancestor prior to the emergence of vertebrates ~ 600 million years ago (Mya)^9,10^. These gene duplications permitted subsequent partitioning of paralogous gene expression between rods and cones and fine-tuning of individual phototransduction components to meet the needs of dim- and bright-light vision. In this way, the duplex retina was established at an early stage of vertebrate evolution^2,8^.

Retinas detect color by comparing the relative activation of multiple cone subtypes, each maximally sensitive to distinct wavelengths. Maximal sensitivity (λ_max_) is primarily determined by the opsin subfamily a cone expresses and the chromophore it contains^5,11^. Prior to the two rounds of whole-genome duplication, four main cone opsin subfamilies emerged via local gene duplication and subsequent molecular diversification: ultraviolet (UV)- (*opn1sw1*; SWS1; range of λ_max_ = 360–420 nm), blue- (*opn1sw2*; SWS2; λ_max_ = 400–470 nm), green- (*opn1mw*; RH2; λ_max_ = 460–510 nm) and red-sensitive opsins (*opn1lw*; LWS; λ_max_ = 510–560 nm)^12,13^. Exclusive expression of one of these opsin subfamilies is the defining characteristic of UV, blue, green, and red cones in many vertebrate species.

Teleost fishes occupy a wide diversity of aquatic habitats and have expanded their opsin repertoires to adapt to these diverse photic niches. Zebrafish (*Danio rerio*) is widely used as a model system in photoreceptor research^14,15^. While the zebrafish genome encodes a single UV cone opsin gene (*opn1sw1*) and a single blue cone opsin gene (*opn1sw2*), it contains a syntenic array of four green cone opsins tuned to a range of wavelengths (*opn1mw1*, λ_max_ = 467 nm; *opn1mw2,* λ_max_ = 476 nm; *opn1mw3,* λ_max_ = 488 nm and *opn1mw4,* λ_max_ = 505 nm), as well as a tandem array of two red cone opsin genes (*opn1lw1*, λ_max_ = 558 nm; *opn1lw2,* λ_max_ = 548 nm)^16^. Prior studies showed differential expression of these green and red cone opsin paralogs across the retina and over developmental time^17^, but the physiological role unique to each individual opsin paralog remains largely unknown. More broadly, a teleost-specific whole-genome duplication occurred ~ 350 Mya at the origin of the teleost lineage^18^. Similar to the genome duplications that occurred earlier in vertebrate evolution^9,10^, this additional teleost whole-genome duplication produced numerous paralogous pairs of photoreceptor-expressed genes, but the expression pattern of these genes among zebrafish photoreceptor subtypes remains largely unknown.

Cellular identity is determined by the combinatorial expression of transcription factors and their cofactors. The multiplicity of cone photoreceptor subtypes in the zebrafish retina makes this species an ideal model for understanding how transcriptional regulators control the development and diversification of closely related, but distinct cell types. Previous studies have identified multiple transcription factors required for vertebrate photoreceptor development and function. In mammals, many transcriptional regulators play a role in both rod and cone development (OTX2, CRX, RAX, MEF2D, and NEUROD1)^19–21^. Whereas others are more specifically involved in rod (RORB, NRL, NR2E3, ESRRB, CASZ1, and SAMD7)^22–27^ or cone development and/or function (THRB, RXRG, RORA, COUP-TFI/COUP-TFII, and OC1/OC2)^28,29^. In zebrafish, studies have begun to identify additional transcription factors required for the development of specific cone subtypes: *tbx2b* in UV cones^30^, *foxq2* in blue cones^31^, *six6a*, *six6b*, and *six7* in blue and green cones^32^, and *thrb* in red cones^33,34^. Despite these advances, the architecture of the transcriptional regulatory networks that govern photoreceptor diversification in zebrafish remain largely unknown. Given the sophistication and complexity of their photoreceptor systems, it is likely that many additional transcriptional regulators remain to be discovered in fish.

Here, we used single-cell RNA-seq to profile adult zebrafish photoreceptors. We identified unique subpopulations of green and red cones in the ventral retina which express red-shifted opsin paralogs and share a specialized complement of phototransduction genes. In addition, we found that other cone subtypes differentially express phototransduction gene paralogs which arose either during the teleost-specific genome duplication or later in specific teleost sub-lineages. Lastly, we discovered numerous transcriptional regulators associated with differential gene expression across zebrafish photoreceptor subtypes; many of these factors were not previously known to be associated with photoreceptor gene regulation.

## Results

### Single-cell transcriptome profiling of adult zebrafish photoreceptors

To reveal the extent of gene expression diversity among adult zebrafish photoreceptor subtypes, we generated single-cell transcriptome data using a droplet-based approach (10 × Chromium single-cell RNA-seq). We obtained enriched populations of photoreceptor cells for our analysis by using two transgenic zebrafish, *Tg(rho:EGFP)ja2Tg* and *Tg(gnat2:EGFP)ja23Tg*, which express GFP in rods and all cone subtypes, respectively^35,36^. GFP-positive cells from each line and a small percentage of GFP-negative cells were isolated via fluorescence-activated cell sorting (FACS) and subjected to single-cell RNA-seq (scRNA-seq) analysis (Fig. 1B). To enhance our ability to detect photoreceptor-specific transcripts, we updated the existing transcript annotation with a publicly available transcriptome derived from adult zebrafish eye (see “Methods”). We used this updated annotation for all of our analyses. We then subjected the scRNA-seq data to multiple rounds of clustering, filtering, and selection to identify 2186 high-quality cells for subsequent bioinformatic analysis. In addition to photoreceptors, we included bipolar cells in our analysis to serve as an ‘outgroup’, since bipolar cells are the cell type most closely related to photoreceptors at the level of gene expression^37,38^.

Unsupervised clustering of the scRNA-seq data categorized cells into eight distinct populations, including five canonical photoreceptor subtypes (UV, blue, green, and red cones and rods) defined by their enriched expression of individual opsin genes: UV cone opsin (*opn1sw1*)*,* blue cone opsin (*opn1sw2*)*,* green cone opsins (*opn1mw1, opn1mw2,* and *opn1mw3*)*,* red cone opsin (*opn1lw2*), and rod opsin (*rho*) (Fig. 1C,D and Fig. S1A). Unsupervised clustering also identified an additional cone population consisting of a mixture of *opn1mw4*-expressing green cones and *opn1lw1*-expressing red cones (Fig. 1D and Fig. S1A). We will discuss this unique *opn1mw4*^+^/*opn1lw1*^+^ population in greater detail below. In addition to these photoreceptor clusters, we identified two populations defined by the expression of the bipolar cell marker genes *cabp5a* and *vsx1*^39,40^ (Fig. 1C,D). These two clusters express genes previously shown to be specific to either mouse ON cone bipolar cells (e.g., *gnao1b, gnb3a, trpm1a, rgs11,* and *isl1*) or rod bipolar cells (*prkcaa*), and OFF cone bipolar cells (e.g.,* fezf2, neto1,* and *zfhx4*) (Fig. 1D, Fig. S1A, and S1B)^41^.

Analysis of differential gene expression among the eight clusters revealed ~ 1100 differentially expressed genes (Fig. 1D, Fig. S1, and Supplementary Data S1). These genes include subtype-defining opsin genes as well as previously identified subtype markers, such as *rom1a* (rod)^42^*, foxq2* (blue)^31^*, thrb* (red)^31^*,* and *si:busm1–57f23.1* (red)^33^. We also identified two pre-microRNAs (mir729 and mir726) expressed exclusively in UV and red cones, respectively, similar to what was previously described in medaka (*Oryzias latipes*)^43^ (Fig. S2). Hierarchical clustering of the top 15 differentially expressed genes from each cluster revealed that a considerable number of genes showed co-expression in multiple cone subtypes (e.g., red + green, UV + blue, etc.) (Fig. S1C). This analysis also showed that genes that are highly specific to single cone subtypes are quite rare (Fig. S1C and Supplementary Data S1) and include *tbx2a* (UV), *grk7b* (UV), *tgfa* (UV)*, mpzl2b* (blue)*, fibcd1a* (green)*, angptl4* (green), and *glis3* (red)*.* To validate these scRNA-seq results, we performed quantitative PCR analysis using reverse transcribed mRNA derived from GFP- or tdTomato-positive subpopulations of rods, cones, and bipolar cells isolated by FACS with lines of transgenic zebrafish (Fig. S3). This analysis confirmed the scRNA-seq results for 24 differentially expressed genes, underscoring the overall validity of our profiling data.

### A unique subpopulation of double cones in the ventral retina

**Figure 2:**
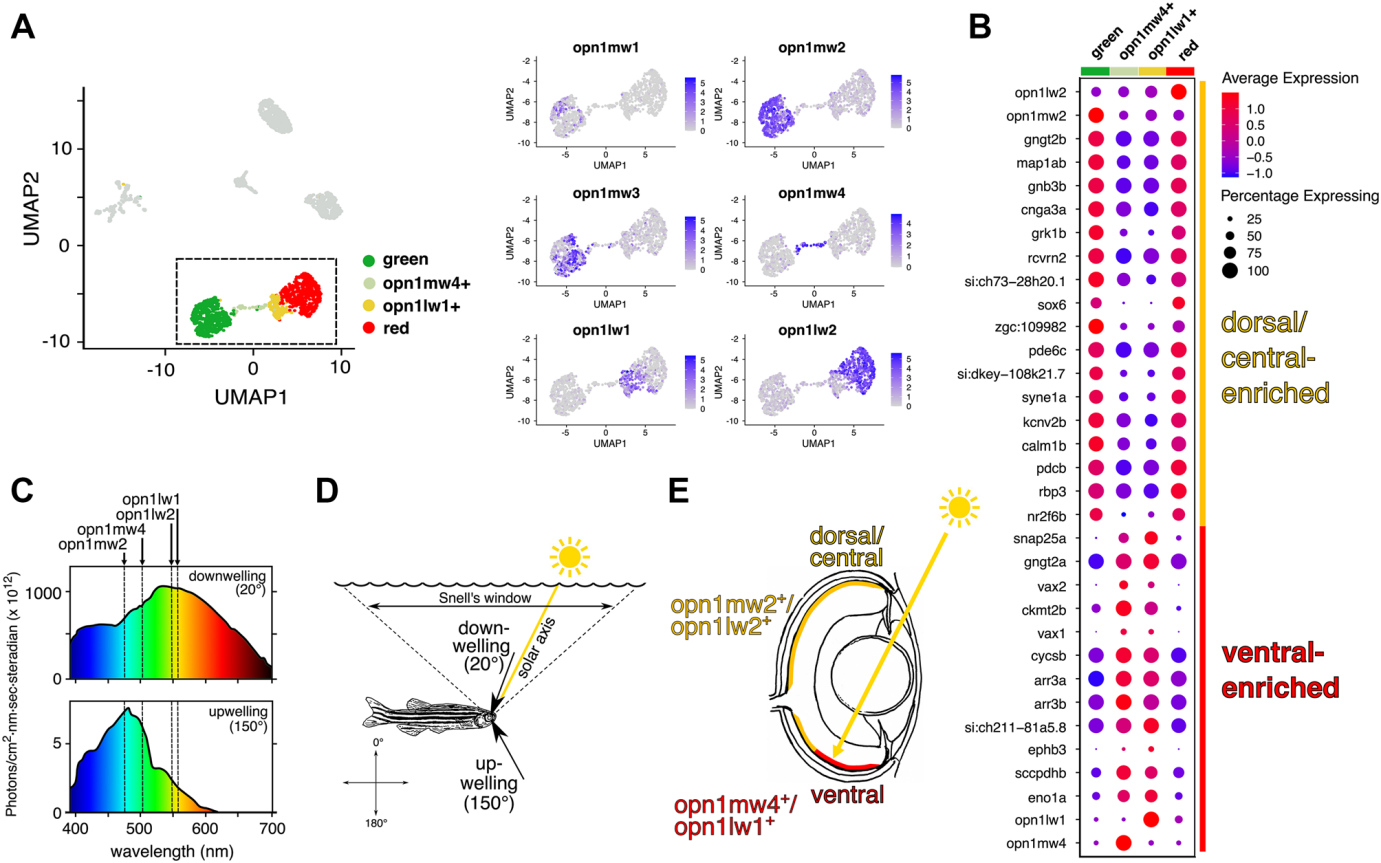
A distinctive subpopulation of red and green cones in the ventral retina. (**A**) Left: UMAP plot of cell clusters from Fig. 1C. Cell clusters except for green and red cones are colored gray. The *opn1mw4*^+^/*opn1lw1*^+^ cells were split into two sub-clusters (*opn1mw4*^+^ and *opn1lw1*^+^) based on the expression of *opn1mw4*^+^ and *opn1lw1*^+^. Right: Expression of green and red cone opsin genes within the cell populations enclosed by the dotted box in the UMAP plot. (**B**) Expression of the top 30 most differentially enriched genes (ranked by adjusted p-value) between ventral (*opn1mw4*^+^ and *opn1lw1*^+^) and dorsal/central (*opn1mw1/2/3*^+^ and *opn1lw2*^+^) green and red cones. Green and red cone clusters were identical to those in (**A**). Dot size reflects the percentage of cells within the cluster expressing the gene, and dot color indicates average expression level within the cluster. (**C**–**E**) Ventrally localized *opn1mw4*^+^ and *opn1lw1*^+^ cones are positioned to detect downwelling light. (**C**) Intensity/spectral distributions for two lines of sight (downwelling light and upwelling light, 20° and 150° from vertical, respectively). These spectra were measured at a depth of 3 m in the lagoon of Enewetok (formerly Eniwetok) Atoll in the Marshall Islands. Data are reproduced from a previous study^62^. The maximum sensitivity of green and red opsin genes are indicated as dotted lines overlying the intensity/spectral distributions^16^. (**D**) From an underwater vantage point, all light from above the water surface enters via a circular aperture known as Snell’s window, which subtends an angle of ~ 96° relative to the fish’s eye irrespective of depth. Scattering and absorption by water cause the dominant wavelengths of transmitted light to vary with the direction of the line of sight. (**E**) The approximate location of the *opn1mw4*^+^ and *opn1lw1*^+^ cones is based on a prior in situ hybridization study^17^

In addition to the four canonical cone subtypes, unsupervised clustering identified a unique subpopulation of cones expressing either *opn1mw4* or *opn1lw1* (referred to here as *opn1mw4*^+^/*opn1lw1*^+^). These cells occupied the region between canonical green and red cone clusters in the 2D plot produced by uniform manifold approximation and projection (UMAP, Fig. 1C) and were defined as members of a single cluster despite their expression of opsins from two different classes (*opn1mw4* is a green cone opsin and *opn1lw1* is a red cone opsin). In many teleosts including zebrafish, red and green cones form a closely apposed pair referred to as a ‘double cone’^44^. Given that *opn1mw4* and *opn1lw1* are expressed primarily in the ventral retina^17^, our data suggest that *opn1mw4*^+^ and *opn1lw1*^+^ cones together form a unique subtype of double cone with a transcriptional profile distinct from that of the green and red cones which comprise canonical double cones. Indeed, even when we subdivide the *opn1mw4*^+^/*opn1lw1*^+^ population into two sub-clusters (*opn1mw4*^+^ and *opn1lw1*^+^) based on opsin expression, we see that they share a distinctive combination of genes (Fig. 2A,B). Compared to the *opn1mw1/2/3*^+^/*opn1lw2*^+^ population, *opn1mw4*^+^/*opn1lw1*^+^ cones are enriched for three genes known to be expressed in the ventral retina: the phototransduction gene *gngt2a*^45^ and the transcription factors *vax1* and *vax2*^46^ (Fig. 2B). The expression of multiple ventrally expressed genes strongly suggests that *opn1mw4*^+^ and *opn1lw1*^+^ cones are localized to the ventral retina. The transcriptomes of *opn1mw4*^+^ and *opn1lw1*^+^ cones also show depletion of various genes, including multiple phototransduction components (*gnb3b, pde6c, gngt2b, rcvrn2,* and *cnga3a*), relative to canonical green and red cones (Fig. 2B). Lastly, *opn1mw4* and *opn1lw1* have the most red-shifted spectral sensitivity (λ_max_) of all green and red cone opsins encoded in the zebrafish genome (Fig. 2C)^16^. These opsins are well-adapted for detecting downwelling light which has a broader and more red-shifted spectral distribution than sidewelling or upwelling light (Fig. 2D,E). The potential functional significance of *opn1mw4*^+^ and *opn1lw1*^+^ cones is discussed in greater detail in the “Discussion”. Overall, these results show that photoreceptor subpopulations may be defined by region-specific gene expression signatures that supersede overly simplistic classifications based on opsin expression alone.

### Partitioning of teleost-specific phototransduction gene paralogs among photoreceptor subtypes

**Figure 3:**
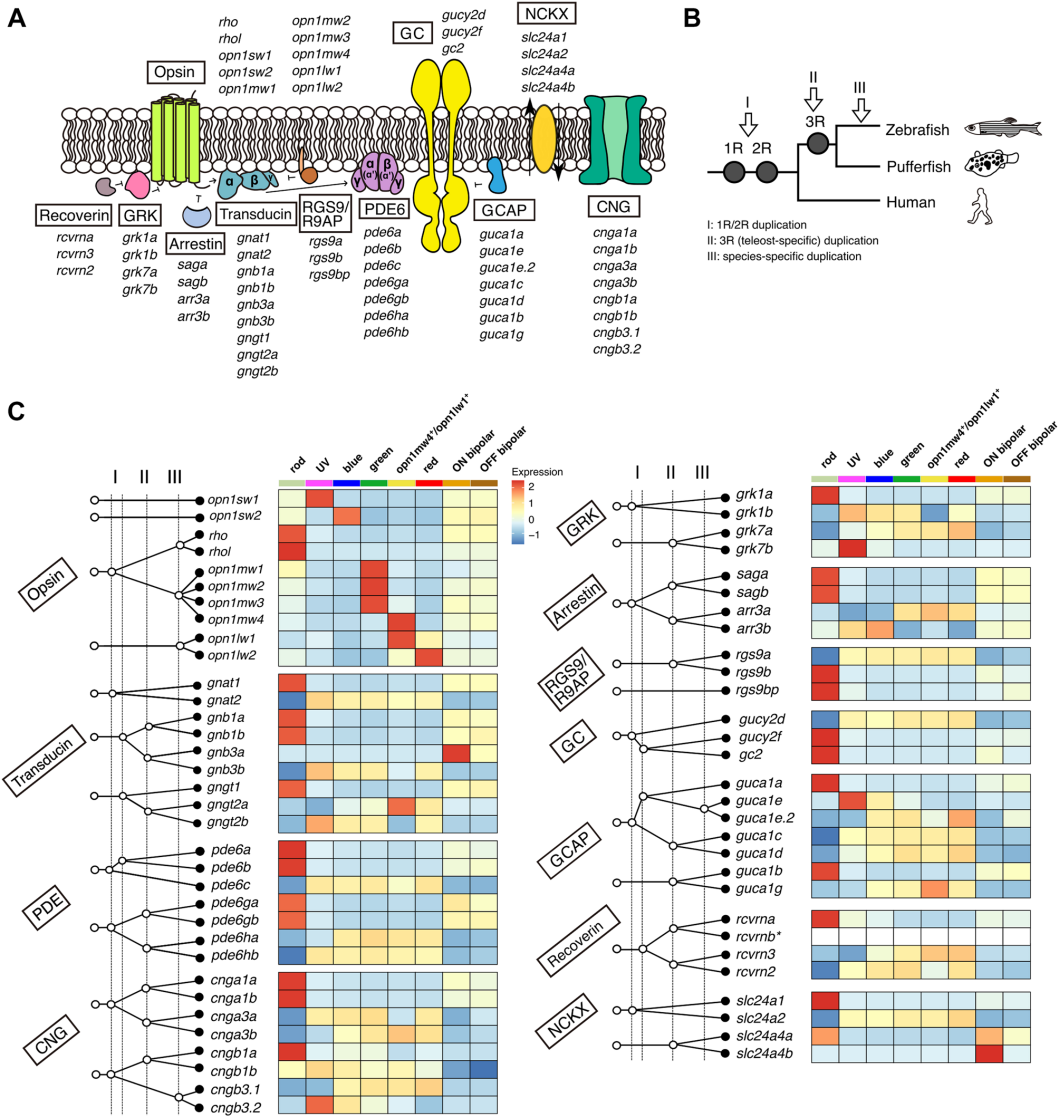
Expression partitioning of paralogous phototransduction genes. (**A**) Schematic representation of vertebrate phototransduction cascade components. During phototransduction, light-activated opsin induces the detachment of the catalytic subunit Gα of the heterotrimeric G protein (transducin) from the inhibitory β/γ subunits. The activated Gα subunit then binds to the two inhibitory γ subunits of cGMP phosphodiesterase 6 (PDE6), thereby relieving inhibition on the catalytic subunits (α, β, and α′). The activated PDE subunits, in turn, catalyze the hydrolysis of the second messenger cGMP, leading to closure of cGMP-gated channels (CNG) on the plasma membrane and photoreceptor membrane hyperpolarization. Shut-off of the activated transducin is accelerated by a GTPase-activating protein complex (RGS9 and R9AP). The light-activated opsin is quenched via phosphorylation mediated by visual pigment kinases (GRK) and by the subsequent binding of arrestins. The activity of GRKs is regulated by binding of recoverin in a calcium-dependent manner. In the recovery/adaptation process, guanylyl cyclase activating protein (GCAP) enhances the synthesis of the second messenger cGMP through guanylyl cyclase (GC) in a calcium-dependent manner. Na^+^/Ca^2+^, K^+^ exchanger (NCKX) is involved in maintaining the dynamic equilibrium of calcium ions in the outer segment. In cones, the ion channel (CNG) and exchanger (NCKX) are located in the plasma membrane, whereas in rods they are located in the disc membrane. Figure design is adapted from Larhammar *et al*., 2009^80^. (**B**) Phylogenetic tree showing the approximate time points at which various genome duplications occurred. (**C**) Evolutionary scenario for gene duplications of vertebrate phototransduction cascade genes (Left panels) and heatmap showing their expression levels in each cell population (Right panels). Left: The four dotted vertical lines mark the events, ‘1R’, ‘2R’, ‘II’, ‘III’, described in (**B**). The horizontal axis is not to scale. White circles indicate putative ancestral genes. Black circles indicate genes encoded in the zebrafish genome. Evolutionary branching patterns for each gene family are described according to the described previous studies^8,45,47,48^ and our BLAST searching results. Figure design is adapted from Lamb, 2020^8^. Right: heatmap showing average expression levels of phototransduction genes in each cluster. Values are row-wise Z-scored gene-expression values. *rcvrnb* expression is not detected.

Expression partitioning of phototransduction gene paralogs between rods and cones occurred early in vertebrate evolution^8^ and mediates key functional differences between these cell classes. The extent to which such partitioning occurs among cone subtypes is currently unknown. Yet, the unique combination of phototransduction genes expressed by *opn1mw4*^+^ and *opn1lw1*^+^ cones suggests that other cone subtypes might also differ with respect to the expression levels of phototransduction genes. We therefore examined the expression patterns and evolutionary origins of all phototransduction-related genes in the zebrafish genome. Guided by the published literature^8,45,47,48^, we identified a total of 63 phototransduction genes, including opsins (Fig. 3A), and found that 46 of these 63 genes arose either during the teleost-specific whole-genome duplication (3R) or during clade- or species-specific gene duplication events after 3R (Fig. 3B,C). The remaining 17 phototransduction genes arose during earlier vertebrate genome duplications (1R or 2R) or before.

Inspection of gene expression patterns reveals that 59 out of 63 phototransduction genes are differentially expressed among photoreceptor and bipolar populations (Fig. 3C, Fig. S4, and Supplementary Data S1). As expected, we found differentially expressed gene ‘pairs’ between rods and cones (*gnat1 *vs.* gnat2, gnb1a/gnb1b *vs.* gnb3b, pde6a/pde6b *vs.* pde6c, pde6ga/pde6gb *vs. *pde6ha/pde6hb, cnga1a/cnga1b *vs.* cnga3a/cnga3b, cngb1a *vs.* cngb3.1/cngb3.2,* and *saga/sagb *vs. *arr3a/arr3b*). All of these gene pairs arose during early vertebrate whole-genome duplications, and their differential expression between rods and cones is conserved between fish and amniotes (e.g., mice and chickens)^37,49^.

We also identified extensive expression partitioning among cone subtypes. All differentially partitioned genes except for *opn1sw1* and *opn1sw2* arose during the teleost-specific whole-genome duplication (*arr3a/arr3b, cnga3a/cnga3b, grk7a/grk7b, gngt2a/gngt2b,* and *rcvrn2/rcvrn3*) or later (*opn1mw1/2/3/4, opn1lw1/2, guca1e/guca1e.2,* and *cngb3.1/cngb3.2*) (Fig. 3C). We noted above the suite of phototransduction genes (*cnga3a/cnga3b, gngt2a/gngt2b,* and *rcvrn2/rcvrn3*) differentially expressed between ventral (*opn1mw4* and *opn1lw1*) and dorsal/central (*opn1mw1/2/3* and *opn1lw2*) green and red cones (Fig. 2B). Additionally, we found multiple pairs of paralogous genes that were differentially enriched between UV cones (*grk7b*, *cngb3.2*, and *guca1e*) and other cone types (*grk7a*, *cngb3.1*, and *guca1e.2*). We also detected partitioning of cone arrestin paralogs between UV and blue cones (*arr3b*) and green and red cones (*arr3a*), as previously described^50^. On the other hand, four pairs of paralogous rod phototransduction genes (*cnga1a/cnga1b, gnb1a*/*gnb1b, pde6ga/pde6gb,* and *saga/sagb*), which arose during the teleost-specific whole-genome duplication, are both expressed in rods. The expression of *rgs9a/rgs9b,* another pair of genes that arose during the teleost-specific duplication, is partitioned between rods (*rgs9b*) and cones (*rgs9a*). Finally, we found that two teleost-specific cone-type transducin β genes, *gnb3a* and *gnb3b,* were partitioned between cones (*gnb3b*) and ON bipolar cells (*gnb3a*). In contrast, the single *Gnb3* ortholog in mouse and chicken is expressed in both cones and ON bipolar cells^37,49^. The expression patterns of several phototransduction gene pairs were confirmed by RT-qPCR analysis of FACS-isolated photoreceptor subtypes and bipolar cells (Fig. S3). In summary, single-cell transcriptome profiling and phylogenetic analysis demonstrate extensive expression partitioning of cone-expressed genes that arose during the teleost-specific whole-genome duplication or later. These differences may mediate differential tuning of the light response in these individual cone subtypes.

### Transcriptional regulatory networks in zebrafish photoreceptors

**Figure 4:**
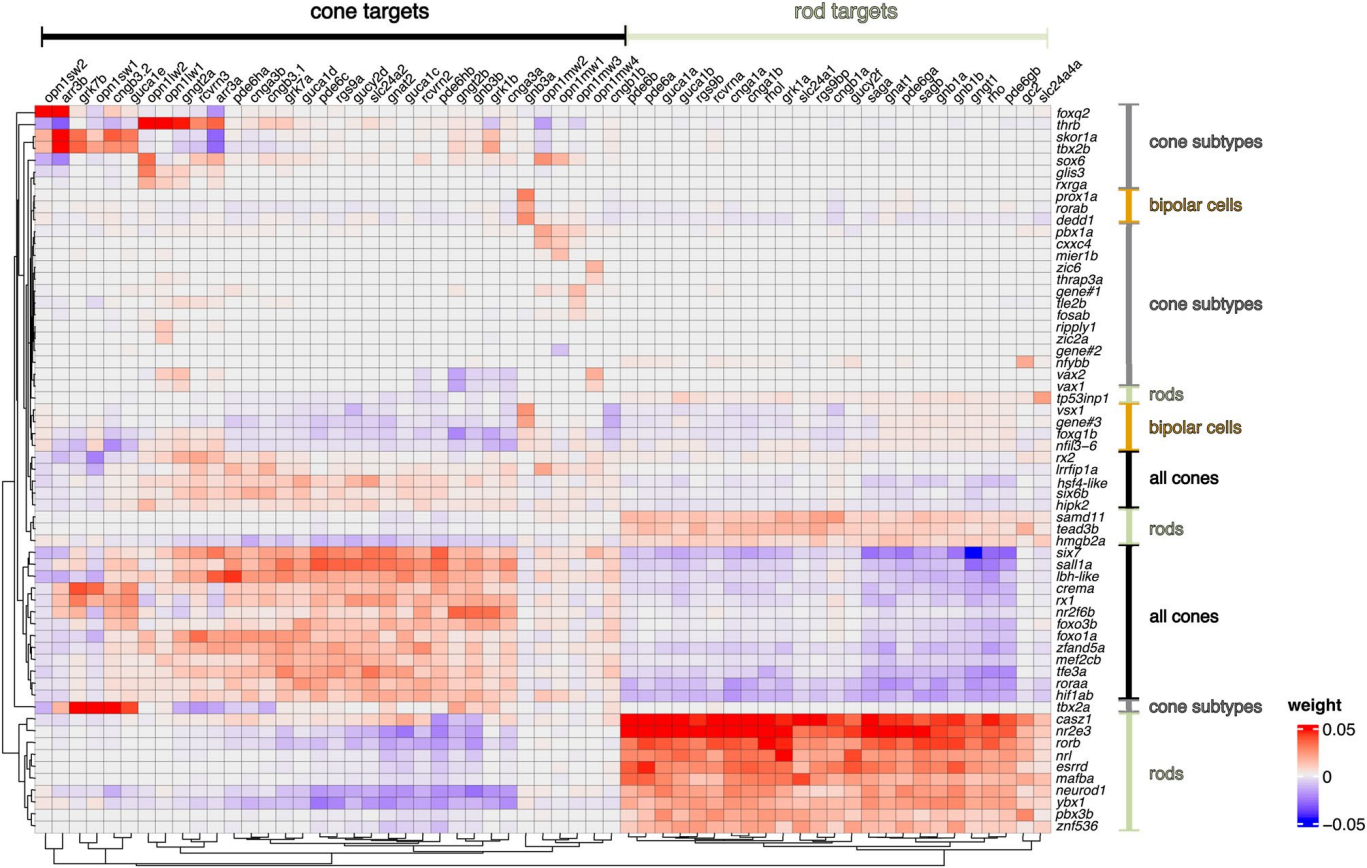
Candidate transcriptional regulators responsible for expression of phototransduction genes. Heatmap showing positive (red) and negative (blue) associations between transcriptional regulators (transcription factors and cofactors) and differentially expressed phototransduction genes (target genes) calculated by GENIE3 algorithm in SCENIC. Rows and columns are arranged according to divisive hierarchical clustering (dividing clusters in a top-down manner). The (dis)similarity of observations was calculated using Euclidean distances. Cell type expression patterns of the transcriptional regulators are presented in Fig. S5A. Gene#1: zgc:114046; Gene#2: zgc:110269; Gene#3: si:ch211–288g17.3.

Transcription factors, cofactors, and chromatin regulators play crucial roles in controlling cell fate and regulating gene expression. To elucidate the relationship between transcriptional regulators and their target genes in zebrafish photoreceptors, we employed a machine learning-based approach, GEne Network Inference with Ensemble of trees (GENIE3) in SCENIC^51,52^. GENIE3 calculates weight scores for each transcriptional regulator (out of a total of 1932), measuring its respective relevance for predicting the expression of each of 59 differentially expressed phototransduction genes (Fig. 4 and Fig. S5). In the following paragraphs, we highlight those transcriptional regulators most strongly implicated in control of phototransduction gene expression by this approach (see “Methods”).

We identified a total of 61 different transcriptional regulators associated with expression of phototransduction genes (Fig. 4). These regulators can be roughly categorized by cell class based on the expression pattern of genes they control: bipolar cells (7 regulators), rods (14), all cones (18), and cone subtypes (22). Our analysis ‘rediscovered’ most rod and cone transcriptional regulators known from previous studies in zebrafish and other vertebrates. In addition, it nominated multiple regulators not previously implicated in photoreceptor gene regulation. Among regulators of rod genes, *casz1*, *nr2e3*, *rorb*, and *nrl* showed the strongest positive association with rod-specific target genes. Two of these genes (*nr2e3* and *nrl*) are known to be essential for rod development in both mice^23,24^ and zebrafish^53,54^, and mouse orthologs of *casz1* and *rorb* are important in photoreceptor gene regulation^22,26^. Other known photoreceptor transcriptional regulators positively associated with rod gene targets include *neurod1*, *samd11*, and *esrrd* (related to mouse *Esrrb*)^25,55,56^. Regulators not previously associated with rod gene expression include *hmgb2a, mafba*, *pbx3b*, *tead3b*, *tp53inp1*, *ybx1*, and *znf536*.

We also identified numerous transcriptional regulators broadly associated with gene expression across cone subtypes or within specific subtypes. Known regulators active in multiple cone subtypes include: *six6b*, *six7*, *roraa, rx1* (*rax2a*), *rx2* (*rax2b*), and *rxrga*. The first two genes (*six6b* and *six7*) were previously shown to be required for development of blue and green cones in zebrafish^32,36^, and orthologs of the latter four have all been implicated in photoreceptor development in birds or mammals^19,57,58^. Novel potential cone regulators include: *crema, foxo1a, foxo3b, hif1ab, hipk2, hsf4-like*, *lbh-like*, *lrrfip1a, mef2cb*, *sall1a, tfe3a,* and *zfand5a*. Our analysis also implicated 22 transcriptional regulators in the control of cone subtype-specific gene expression. Known regulators such as *tbx2b*, *foxq2*, and *thrb* show the strongest positive associations with UV, blue, and red cone-specific marker genes, respectively^30,31,34^. We also discovered novel candidate regulators of UV cone genes (*tbx2a*), UV and blue cone genes (*skor1a*), and red cone genes (*sox6* and *glis3*). Additionally, we identified *vax1* and *vax2* as putative regulators in *opn1mw4*^+^ and *opn1lw1*^+^ cones; these two factors showed strong positive associations with the target genes: *opn1mw4, opn1lw1,* and *gngt2a*. The tandemly arrayed cone opsin genes (*opn1mw1/2/3/4* and *opn1lw1/2*) were positively, but weakly, associated with several regulators (*cxxc4*, *fosab, mier1b*, *pbx1a*, *ripply1*, *tie2b*, *thrap3a*, *zic2a*, and *zic6*).

We next used SCENIC to perform gene regulatory network analysis with a set of differentially expressed non-phototransduction target genes (Fig. S5 and S6, see “Methods” for further detail). We identified many of the same transcriptional regulators that we found for phototransduction gene targets. In addition, we found additional known regulators of rod and cone gene targets, including *six6a* and *otx5* (both positively associated with cone gene expression). CRX, an ortholog of *otx5*, plays a critical role in photoreceptor gene regulation in mammals^21^, and *six6a* (along with *six6b* and *six7*) was previously shown to be required for blue and green cone gene expression in zebrafish^32^. This new analysis also highlights multiple additional regulators that are weakly associated with cone gene expression: *cbx4*, *hlfa*, *hmga1b*, *hsf1*, *meis1b*, *mlf1*, *mn1a*, *nr2f1b*, *phf19*, *rxrba, si:ch73-386h18.1*, *ss18l2*, *top1l*, *tsc22d3*, *tshz3a*, and *xbp1*. Collectively, these analyses highlight many transcriptional regulators of photoreceptor gene expression known from prior studies in zebrafish and other vertebrates and reveal a wide range of novel factors potentially involved in the regulation of rod-, pan-cone-, or cone-subtype-specific gene expression.

## Discussion

In the present study, we used scRNA-seq to profile adult zebrafish photoreceptors. We identified a distinctive subpopulation of green and red cones concentrated in the ventral region of the retina, which expresses red-shifted opsin paralogs and a unique complement of phototransduction genes. We also found that canonical UV, blue, green, and red cone subtypes differentially express paralogous phototransduction genes, which arose either during the teleost-specific genome duplication or later. Lastly, we discovered numerous transcriptional regulators associated with differential gene expression across zebrafish photoreceptor subtypes. This work lays a foundation for future studies aimed at understanding how molecular differences among cone subtypes affect photoreceptor function.

Early vertebrate whole-genome duplications (1R and 2R) provided the raw genetic material for the subsequent evolution of distinct rod- and cone-specific opsins and phototransduction components. Similarly, we find that zebrafish cone subtypes show differential expression of many phototransduction genes, but in this case, nearly all differentially expressed paralog pairs appear to have arisen during the teleost-specific whole-genome duplication (3R) ~ 350 Mya or later. This finding suggests that partitioning of paralogous gene expression may be a common mechanism of cell type diversification that permits physiological fine-tuning of serially ‘paralogous’ cell types. Interestingly, additional whole-genome duplication events have occurred in teleosts: in the common ancestor of salmonids (~ 80 Mya)^59^ and in the common ancestor of goldfish (*Carassius auratus*) and the common carp (*Cyprinus carpio*) (~ 14 Mya)^60^. Future analyses of differential gene expression among photoreceptor subtypes in salmon or goldfish may reveal whether expression partitioning of paralogous phototransduction components invariably follows genome duplication and how rapidly it occurs in evolution.

We observed distinct patterns of opsin and phototransduction gene expression between dorsal/central double cones (*opn1mw1/2/3* and *opn1lw2*) and ventral double cones (*opn1mw4* and *opn1lw1*) (Fig. 2). The partitioning of gene expression between these two populations suggests that *opn1mw4*^+^ and *opn1lw1*^+^ cones may be specialized for the detection of light with special qualities. In the wild, zebrafish are known to inhabit shallow, slow-moving streams and pools^61^. A classic study of light in a shallow, tropical marine environment showed that downwelling light along the solar axis is more red-shifted, of far greater intensity, and noisier (due to surface turbulence) than light along all other lines of sight^62^ (Fig. 2C–E). This downwelling light is expected to impinge upon the retina in the distribution of the *opn1mw4*^+^ and *opn1lw1*^+^ cones when the fish is in a horizontal position (Fig. 2D,E), suggesting that the expression of red-shifted opsin paralogs in these cells may serve to enhance detection of red-shifted downwelling light. Conversely, it has been proposed that reduction of *gnb3b* expression in the ventral zebrafish retina may have evolved to protect photoreceptors from high-intensity downwelling light by decreasing the gain of the phototransduction cascade^45^. In either case, our findings reveal a complex suite of changes in the expression of phototransduction genes in ventral cones, suggesting the existence of functional adaptations at multiple levels of the phototransduction cascade.

We also found expression partitioning of 3R-duplicated phototransduction genes among canonical cone subtypes, most notably between UV cones and non-UV cones (Fig. 3). The functional role of this distinctive gene expression signature in adult UV cones is currently unknown, but a prior study of larval zebrafish retina found distinctive gene expression in UV cones within the ‘strike zone’, a region of the ventral-temporal retina specialized for the detection of UV-reflective prey^63^. The adult UV cone-enriched paralogs are all involved in calcium-mediated feedback regulation of the phototransduction shut-off cascade and recovery/adaptation in cones (Fig. 3)^8,14,50,64^. Future functional studies will be required to determine the precise functional role of this adult UV cone-enriched gene expression program.

Our analysis of transcriptional regulators in zebrafish photoreceptors implicated dozens of factors in the control of rod, pan-cone, and cone subtype genes (Fig. 4 and Fig. S6). A role for many of these factors in photoreceptor development had been previously demonstrated in other vertebrates, but not in zebrafish. Thus, our findings underscore a striking degree of evolutionary conservation within vertebrate photoreceptor transcriptional networks, extending from fish to mammals. Zebrafish retain the full set of four canonical cone subtypes inferred to have been present in the common ancestor of fish and mammals, while mammals lost two of those cell types (*opn1sw2*-expressing blue cones and *opn1mw*-expressing green cones) in the course of evolution^65^. So-called ‘green’ cones in mice and humans express orthologs of fish *opn1lw* (not *opn1mw*) and therefore arose from ancestral red cones^12^. The retention of the four ancestral cone types in zebrafish makes this species an excellent system for discovering features of vertebrate photoreceptor transcription networks that may have been lost in mammals. In addition, the present study suggests an even greater degree of complexity within the photoreceptor transcription network than previously suspected, revealing many transcriptional regulators not previously implicated in photoreceptor development or differentiation. Some of these novel factors likely play determinative roles in photoreceptor cell fate, whereas others may fine-tune gene expression in more subtle ways.

## Methods

### Zebrafish husbandry

Zebrafish were raised and maintained according to established protocols^66^. All experiments were designed according to the ARRIVE guidelines, carried out in accordance with the Guide for the Care and Use of Laboratory Animals of the National Institutes of Health, and approved by the Washington University in St. Louis Institutional Animal Care and Use Committee (protocol# 19-1110). Adult fish were raised in a 14-h light/10-h dark cycle and fed with dry food once per day and with rotifers twice per day. *Tg(rho:EGFP)ja2Tg*^35^, *Tg(gnat2:EGFP)ja23Tg*^36^, *Tg(-5.5opn1sw1:EGFP)kj9Tg*^67^, *Tg(-3.5opn1sw2:EGFP)kj11Tg*^68^, and *Tg(opn1mw2:EGFP)kj4Tg*^69^ fish were obtained from the Zebrafish International Resource Center and National BioResource Project Zebrafish. *Tg(thrb:Tomato)q22Tg*^34^ was obtained from Dr. Rachel Wong at the University of Washington. *TgBAC(vsx1:GFP)nns5Tg*^70^ was obtained from Dr. Ryan B. MacDonald at University College London.

### Isolation of rod and cone photoreceptors by fluorescence-activated cell sorting (FACS)

For isolation of rod and cone photoreceptors, we used the transgenic zebrafish lines, *Tg(rho:EGFP)ja2Tg*^35^ and *Tg(gnat2:EGFP)ja23Tg*^36^, which express GFP in rods and all cone subtypes, respectively. Five-month-old adult zebrafish were euthanized by submersion in ice water and their retinas were harvested at around zeitgeber time 3 (ZT 3). The dissected retinas were washed twice with calcium- and magnesium-free ﻿Hanks’ balanced salt solution (HBSS). Two retinas were incubated for 10 min at 37 °C in 400 µl of an activated papain dissociation solution (50 mM HEPES, 2.5 mM l-Cysteine, 0.5 mM EDTA, 23 U/ml Papain Suspension [LS0003126, Worthington] in calcium- and magnesium-free HBSS), that had been pre-activated by incubation for 10 min at 37 °C. After papain incubation, the retinas were centrifuged at 1500×*g* for 30 s. The supernatant was removed, and the retinas were further incubated for 5 min at 37 °C in 600 µl of 10% fetal bovine serum (FBS) in Dulbecco modified Eagle medium (DMEM) with 5 mM magnesium and 5 U DNaseI (Cat. No. 04716728001, Roche). The incubated samples were then gently triturated five times with a P1000 pipette to generate a single-cell suspension. The dissociated retinas were then centrifuged at 300×*g* for 5 min. After drawing off the supernatant, the samples were gently triturated again five times with a P1000 pipette in 300 µl of sorting buffer (20 mM HEPES and 0.04% bovine serum albumin in Calcium- and magnesium-free HBSS, pH 7.4). Four retinas from two individuals were combined for *Tg(rho:EGFP)* fish, while six retinas from three individuals were combined for *Tg(gnat2:EGFP)* fish. The combined samples were each passed through a 35 µm nylon mesh filter into a polypropylene FACS tube. Cell viability was evaluated by incubating the cells in a solution of propidium iodide (10 µg/ml) on ice for 5 min before cell sorting. The filtered samples were also incubated in a solution of Hoechst 33342 (5 µg/ml) to label nuclei. GFP-positive cells were isolated with a fluorescence activating cell sorter (FACSAria, BD Biosciences). Cells were initially filtered by forward- and side-scatter signals. Dead cells were then removed based on propidium iodide positivity. Intact rods and cones were then selected based on the presence of both blue (Hoechst 33342) and green fluorescence (GFP). About 35,000 viable, intact GFP-positive cells (PI^-^, GFP^+^, Hoechst^+^) and 1500 GFP-negative cells (PI^-^, GFP^-^, Hoechst^+^) were collected from *Tg(gnat2:EGFP)* fish, while 10,000 viable, intact GFP-positive cells and 1500 GFP-negative cells were collected from *Tg(rho:EGFP)* fish. These isolated cells were collected in 600 µl of the sorting buffer in 1.5 ml microtubes. The collected cells were then centrifuged at 300×*g* for 5 min and the supernatant was removed. Cell density was quantified on a hemocytometer, and ~ 6000 cells were used for sequencing library preparation.

### Assembling an adult zebrafish eye transcriptome

We retrieved publicly available strand-specific RNA-seq data for the adult zebrafish eye (European Nucleotide Archive, ERR4029230)^71^. StringTie (v2.1.4)^72^ was used to assemble a genome-guided transcriptome with an improved annotation file (v4.3.2.gtf)^73^ as an initial guide. RNA-seq reads were mapped onto the reference transcripts in a strand-specific manner using the STAR aligner^74^ with the command-line options --outSAMattrIHstart 0 --outFilterIntronMotifs RemoveNoncanonical --outSAMstrandField intronMotif. Next, StringTie was used with the command-line options (--rf -t -G) to assemble new transcripts based on the RNA-seq reads with the reference annotation file (v4.3.2.gtf) guiding the assembly process. StringTie was then rerun with the command line option (merge) to obtain an updated transcript annotation, which contained both reference transcripts and non-redundant assembled transcripts predicted by the sequencing reads. The novel transcripts were named according to StringTie’s naming convention (e.g., MSTRG.19429). Some of the novel, de novo loci may correspond to non-coding RNAs or enhancer RNAs. We included these ‘genes’ in our analysis to enhance cell clustering. The StringTie merge mode concatenates transcript IDs of multiple genes when those transcripts overlap with each other, and the expression levels of these concatenated genes are counted as a single gene in the 10X Genomics Cell Ranger pipeline. To determine which of the concatenated genes is actually differentially expressed among cell clusters, we manually inspected pseudo-bulk RNA seq reads described in the following section. The gene symbol of the differentially expressed gene was then used to replace the corresponding concatenated name.

### Single-cell RNA-seq

#### Sample preparation and sequencing

Single-cell libraries were prepared using the Chromium v3 platform (10X Genomics, Pleasanton, CA) according to the manufacturer's instructions. Both GFP-positive and GFP-negative cells were collected from adult zebrafish *Tg(rho:EGFP)ja2Tg* and *Tg(gnat2:EGFP)ja23Tg* as described in the previous section, and approximately 6000 single cells were used for library preparation. Single cells were partitioned into Gel beads in EMulsion (GEMs) using the GemCode instrument, followed by cell lysis and reverse transcription of RNA, amplification, shearing, adaptor ligation, and sample index attachment. Libraries were sequenced on an Illumina NovaSeq machine (540 million paired-end reads: Read 1: 28 bp, Read 2: 98 bp). Sample demultiplexing, alignment to the genomic reference (GRCz11), quantification, and initial quality control was performed using Cell Ranger software (version 6.0.0, 10X Genomics). The eye-specific transcript reference assembly described above was used for the alignment of reads. The GFP transcript sequence was added manually to the reference assembly as an extra chromosome. We initially obtained a matrix consisting of 27,931 genes × 12,833 cells. The greater number of the recovered cells (~ 13,000 cells) than expected (~ 6000 cells) suggested that a sizeable fraction of GEMs contain only ambient RNA or organelles such as mitochondria. These ‘cells’ were removed during subsequent data processing.

#### Data processing

Data were analyzed using the Seurat R package (v4.0.0)^75^. We retained all cells that expressed > 500 genes, and we required all genes to be expressed in at least five cells. Cells with greater than 30% mitochondrial gene content (likely representing dead cells) or > 40,000 unique molecular identifiers (likely representing doublets/multiplets) were removed from the analysis. For the remaining cells (8793 cells), a gene expression matrix was normalized to total cellular read counts using the negative binomial regression method implemented in the Seurat SCTransform function with the method set to glmGamPoi. The 3000 most variable genes, identified by the SCTransform function, were used for Principal Component Analysis (PCA). The top 30 principal components were selected for subsequent analysis according to the elbow plot. Graph-based clustering was performed to obtain a set of transcriptionally distinct clusters. At this point in the analysis, we deliberately set parameters to "over cluster" the data, to avoid combining distinct cell types and to identify sub-populations of low-quality cells for removal. In addition to photoreceptor cells, we initially identified several classes of retinal cells such as bipolar cell, horizontal cell, retinal pigment epithelium, and Müller glia. Of these non-photoreceptor cell types, we only retained bipolar cells which were used as an outgroup in our subsequent analyses.

#### Cell clustering and filtering

To retain high-quality photoreceptors and bipolar cells only, we subjected our data to multiple rounds of clustering, filtering, and selection. In the first round, we retained those clusters characterized either by the presence of one or more of the following opsin genes or phototransduction genes (*rho*, *opn1sw1*, *opn1sw2*, *opn1mw1*, *opn1mw2*, *opn1mw3*, *opn1mw4*, *opn1lw1*, *opn1lw2*, *gnat1*, or *gnat2*) or bipolar-specific genes (e.g., *gnao1b, vsx1, cabp2a, cabp5a,* and *cabp5b*) among the top 20 most differentially expressed genes as identified by the FindAllMarkers function in Seurat with the Wilcoxon rank sum test. We also removed clusters consisting of low-quality cells with low total gene counts (500–1000 genes/cell) compared with high-quality photoreceptor clusters (1000–3000 genes/cell for rods and 1000–4000 genes/cell for cones). A total of 2602 cells were retained after the first round of filtering. In the second round of clustering and selection, we removed clusters that showed co-expression of photoreceptor genes and Müller glial genes (*icn*, *fxyd6l*, *mt2*, *rlbp1a*, and *glula*). Previous single-cell studies showed that zebrafish Müller glia often show aberrant photoreceptor gene expression, likely due to adherence of fragments of photoreceptor cytoplasm to the cells^76^. We also removed one cluster showing co-expression of photoreceptor and bipolar genes and with low total gene counts. In the final round of clustering and selection, we removed a rod subpopulation with low total gene counts. A final set of 2186 high-quality cells was used for subsequent bioinformatic analyses.

#### Differential expression test

Genes differentially expressed among clusters were identified using the FindAllMarkers function in Seurat with arguments “test.use = ‘wilcox’, min.pct = 0.25, logfc.threshold = log2(1.5)”. This list of differentially expressed genes is available in Supplementary Data S1. Genes differentially expressed between ON and OFF bipolar cells were identified using the FindMarkers function in Seurat with arguments “test.use = ‘wilcox’, min.pct = 0.1”. Genes differentially expressed between dorsal/central and ventral green/red cones were identified using the same approach used for bipolar cells.

#### Pseudo-bulk average expression profile

The mapped sequence reads (BAM file) were subsetted using cellranger-dna bamslice to generate pseudo-bulk read counts for each cell cluster. The subsetted reads were each counted using subread featureCount v2.0.0^77^ with an improved annotation file (v4.3.2.gtf)^73^. Normalized RNA sequencing reads (transcripts per million, TPM) of differentially expressed genes for each cluster are included in the list of differentially expressed genes (Supplementary Data S1).

#### Gene nomenclature

Phototransduction genes were manually curated from the Ensembl database [http://www.ensembl.org/; (Release 104)] according to the literature^8,45,47,48^. Some of these gene names were revised according to NCBI Gene database [www.ncbi.nlm.nih.gov/gene; (cited 2021 May)] as well as on the basis of manual BLAST searches. The revised gene names and their accession numbers are listed in Supplementary Table S1.

### Transcriptional regulatory network analysis

We used the SCENIC R package^51^ (v1.2.4) to identify associations between transcriptional regulators and target genes in our datasets. One hundred cells were randomly chosen from each of the eight clusters, and standardized gene expression scores (scale.data in Seurat) derived from the total set of 800 cells were used for the analysis. A list of transcription factors, transcription cofactors, and chromatin regulators was retrieved with ZebrafishMine using the following gene ontology terms: “negative regulation of transcription, DNA-templated”, “regulation of transcription, DNA-templated”, “positive regulation of transcription, DNA-templated”, “DNA-binding transcription factor activity, RNA polymerase II-specific”, “RNA polymerase II cis-regulatory region sequence-specific DNA binding”, and “DNA binding”. We retained transcription factor/cofactor/chromatin regulators that were detected in at least 1% of the cells and which were represented by at least eight transcripts (normalized for each cell by the total expression and multiplied by a scale factor, 10,000) in total across all samples, yielding a total of 1932 genes. Phototransduction genes (target genes) were manually curated from Ensembl genome and/or NCBI databases according to the literature^8,45,47,48^. We only retained 59 phototransduction genes, which are included in the list of differentially expressed genes among clusters (Supplementary Data S1).

For the analysis of non-phototransduction-related target genes, we retained the top 10 differentially expressed genes for each photoreceptor cluster (Supplementary Data S1) after excluding both phototransduction genes and transcriptional regulatory genes. The GENIE3 algorithm^52^ was implemented in SCENIC to generate random forest weights of transcriptional regulators for each target gene. Weights reflect the predictive power of each regulator in determining the expression level of each target gene. In parallel, Spearman correlation coefficients between regulators and target genes were calculated using the runCorrelation function. To indicate whether a transcriptional regulator had an activating or repressive effect on a target gene’s expression, we preserved the sign of the Spearman correlation coefficient (i.e., a positive coefficient indicates an activating effect and a negative coefficient indicates a repressive effect). We used the “top5” cutoff in SCENIC to only display the strongest regulatory linkages identified by the algorithm. The selected transcriptional regulators and target genes were clustered using the Heatmap function in ComplexHeatmap^78^ with hierarchical clustering of the weights for visualization. We used customized versions of some SCENIC functions, regarding geneFiltering and runGenie3 to allow use of our gene lists. The custom scripts were made referring to the previous study^79^.

### RT-qPCR validation of single-cell profiling results

Ages and genomic features for each transgenic fish used for RT-qPCR are described in Supplemental Table S2. Dissociated retinal cells were prepared for each transgenic fish as described in the section above, but without propidium iodide and Hoechst 33342 staining. Cells were filtered by forward- and side-scatter signals, and then 10,000 GFP- or tdTomato-positive were collected into 300 µl of lysis buffer (Buffer RL, Norgen Biotek Corporation) in 1.5 ml microtubes. Total RNA was extracted with a Single Cell RNA purification kit (Norgen Biotek Corporation). The extracted RNA was reverse-transcribed with SuperScript IV (Invitrogen) and oligo(dT) primers according to manufacturer’s instructions. The reverse-transcribed cDNA was subjected to quantitative PCR using Power SYBR Green Master Mix (Thermofisher Scientific) and the QuantStudio 3 Real-time PCR system (Thermofisher Scientific) according to manufacturer’s instructions. Expression levels were calculated by the relative standard curve method. The standard curve was prepared with serial dilutions of cDNA samples reverse-transcribed from total RNA of zebrafish eye. The transcript levels were normalized to ribosomal protein L13a (*rpl13a*) transcript levels in all analyses. Primers used for quantitative PCR are listed in Supplemental Table S3.

### Statistical analysis

Sample sizes were determined based on prior literature and best practices in the field. The Tukey–Kramer HSD (honestly significant difference) test was used to determine the statistical significance among multiple datasets (the ‘multcomp’ package v1.4-16 in R, version 4.0.0).

## Supplementary Information

Supplementary Information 1.

Supplementary Information 2.

## Data Availability

The datasets generated during and/or analyzed during the current study are available from the corresponding author on reasonable request. All data generated or analyzed during this study are included in this published article (and its Supplementary Information files). The datasets generated in the current study are available in Gene Expression Omnibus (GSE175929).
