# Supplementary material for: Partitioning of gene expression among zebrafish photoreceptor subtypes
Source: Sci Rep. 2021 Aug 30;11:17340. doi: 10.1038/s41598-021-96837-z (PMC8405809; doi:10.1038/s41598-021-96837-z)
Supplement: Supplementary file 2 — Supplementary Information 2. [file 41598_2021_96837_MOESM2_ESM.pdf]

Supplemental files for:

Partitioning of gene expression among zebrafish photoreceptor subtypes

Yohey Ogawa and Joseph C. Corbo

Dept. of Pathology and Immunology, Washington University School of Medicine, St. Louis, MO

Supplemental Table S1

Revised gene names and accession numbers

gene name	original name*	Gene ID (NCBI or Ensembl)
pde6hb	*zgc:73359*	393810 (NCBI gene ID)
guca1e.2	*si:ch211-103a14.5*	ENSDARG00000100747
cngb1b	*si:dkey-44k1.5*	ENSDARG00000042107
fibcd1a	*CU639468.1-si:dkeyp-51b7.3*	ENSDARG00000074640
mir726	*CT573282.1*	ENSDARG00000082495
hsf4-like	*si:dkey-18a10.3*	ENSDARG00000090814
lbh-like	*si:ch1073-303d10.1*	ENSDARG00000103006

* “original name” is the gene name in the assembled transcript annotation that was used in our scRNA-seq analysis (See Methods).

Supplemental Table S2

Zebrafish lines used for quantitative PCR analysis

Genotype	Age (months)	Reference
Tg(rho:EGFP)ja2Tg	7-8	(1)
Tg(-5.5opn1sw1:EGFP)kj9Tg	10-11	(2)
Tg(-3.5opn1sw2:EGFP)kj11Tg	9	(3)
Tg(opn1mw2:EGFP)kj4Tg	9	(4)
Tg(thrb:Tomato)q22Tg	18	(5)
TgBAC(vsx1:GFP)nns5Tg	10	(6)

Supplemental Table S3

Primers used for quantitative PCR

Target gene	Forward (5' to 3')	Reverse (5' to 3')
rho	ACTTCCGTTTCGGGGAGAAC	GAGTGCGGGTGTAGTAGTCG
opn1sw1	CGAGAGATATGTGGTCATCTG	TGTATCTGCTCCATCCAAAG
opn1sw2	GGAGGAATGGTGAGTTTGTG	GGTCTTGAAGGTAAAGTTCC
opn1mw2	GCGTGGGTAGATTAGTTGTG	GGCTTATGCTCAGATTTAGTGG
opn1lw1	TTATGTCTTCATGAACCGACAG	ATTTTCATCTTTTTCCCCATGTC
rpl13a	TCCCAGCTGCTCTCAAGATT	ACTTCCAGCCAACTTCATGG
angptl4	GAGATGACACCCGAAGGAGG	GCCACCTTTGGACACAGAATG
cabp5a	GCCAAAATTGACCGAGAGCTGG	CAGGTTCCCCAGATCCTTACAG
si:busm1-57f23.1	GCTAGCACTCAGGTTACAAATAGG	CCATGGTGACCTCAAACTTGC
mpzl2b	TAAATCCGCCCGACATCCAG	CCAGAATCCCGATTTCAGAG
tbx2a	ACTGGACCAGCAACAAGTCC	GTCCTTGACTCTGTCCTCGC
gnb3a	ATGCATTGGGGGACAGACTC	TTGCATGCACCTTATTGGTGG
gnb3b	TGCTCACACAGGTTACTTGTCC	AGTCTCCAAATCCCACAGTGC
grk1a	GAGACCTCAAGCCGGAGAAC	GCGCCATGAACCCTGGAG
grk7a	GAGCACAAGAACTTCGACGC	ATTAGTCCAGCCTCCAAGCG
grk7b	GGACCGCCTTGGCAGC	TGTCACCTGTGTCCTTAGCG
rcvrn2	CTGCCAGGCTATCTTTAAGCTG	TAAACTCGCCCTCAGCCAAT
rcvrn3	CTAAAGCTGGAGTGGGCCTT	TTTTCTCTGGCGTGCTCTCA
gngt1	CGTAAGCAGCAAAAATGCCG	GGATCTTCATCCGCTCCTCC
gngt2a	GTGACCTGTTGCCTCCATCG	TTTAGAGACAGGCTCTCTGGT
gngt2b	ACACACTTCTCTGACTGCCG	TTGCTGAAACCGCGGTACG
guca1a	CTGGATCCAAAAGCCAATGCC	CGGCCACGTACTCCATAAAG
guca1c	TCACGTTTGACATGGACGGG	CCTGGATGGCCTTGAATATGGT
guca1e	GCTCTTCGATATGGACGGCA	GACAACTCGCCGTCTCCATT
guca1e.2	AGCTGCGCTGGTATTTCAAG	ACACTCCGTCTGCATTGAGG

Supplemental figure

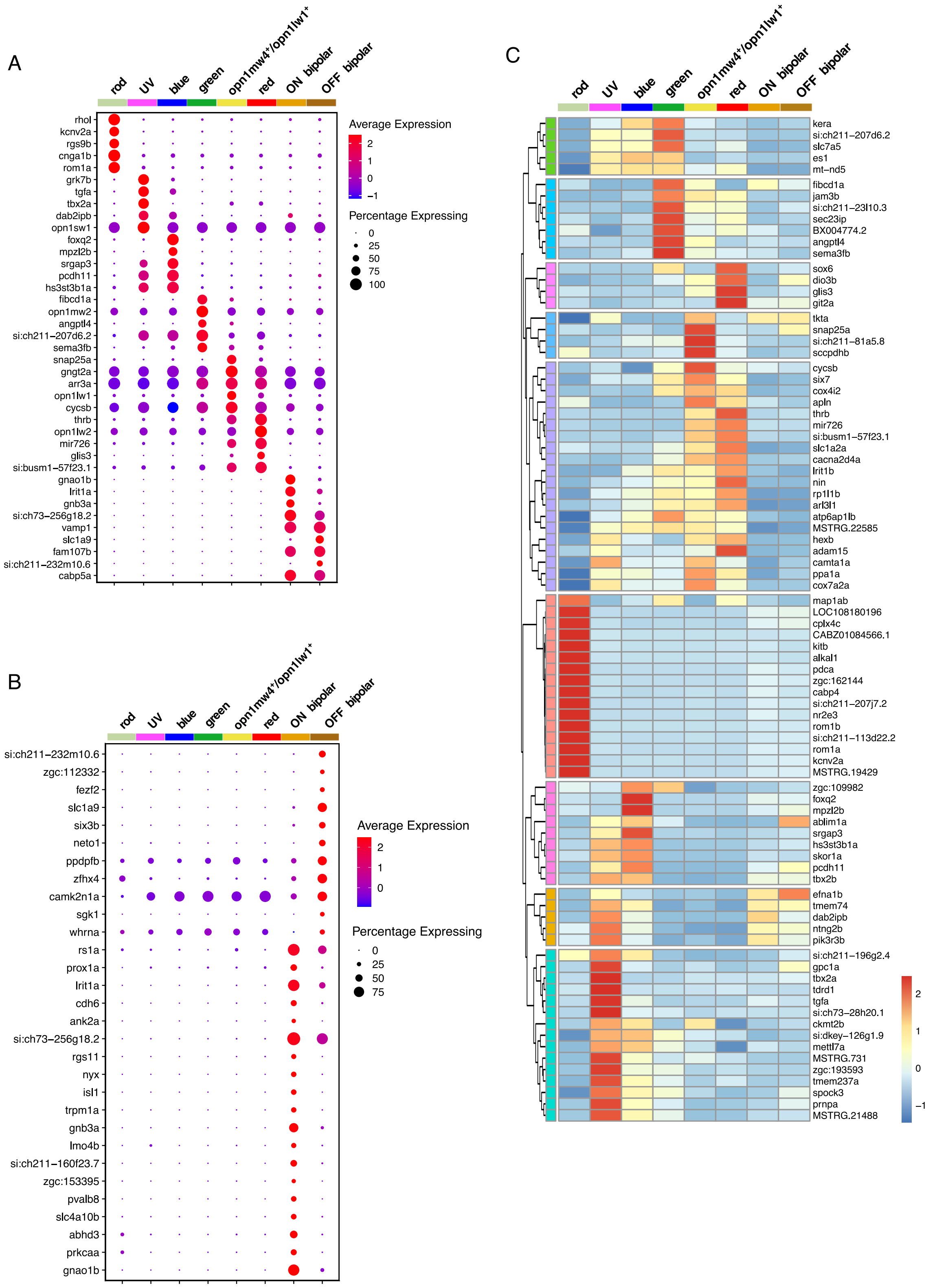

Figure S1. Expression patterns of cluster-enriched marker genes and phototransduction genes

(**A**) Dot plot showing the expression patterns of the top five differentially enriched genes for each cluster. Genes were ranked by adjusted p-value and grouped by cell cluster. Dot size reflects the percentage of cells within the cluster expressing the gene, and dot color indicates average expression level within the cluster. Values are row-wise Z-scored gene-expression values. Note that our single-cell data shows evidence of cross-cluster contamination with ambient RNA from several genes, most notably *opn1sw1*, *arr3a,* and *gngt2a*. (**B**) Dot plot showing the expression patterns of the 30 most differentially enriched genes between ON and OFF bipolar cells. (**C**) Heatmap showing average expression levels of the top 15 differentially expressed genes. Phototransduction isoform genes were excluded in this analysis. Values are row-wise Z-scored gene-expression values. The marker genes were arranged by hierarchical clustering with the 'complete' agglomeration method using the average gene expression levels.

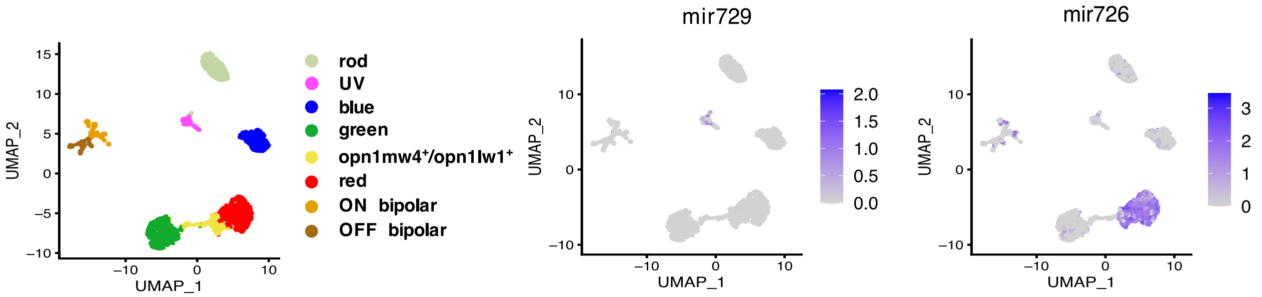

Figure S2. Expression patterns of pre-microRNA genes

Left: UMAP plot of cell clusters from Fig. 1C, reproduced here for reference. Right: Expression patterns of pre-microRNA genes.

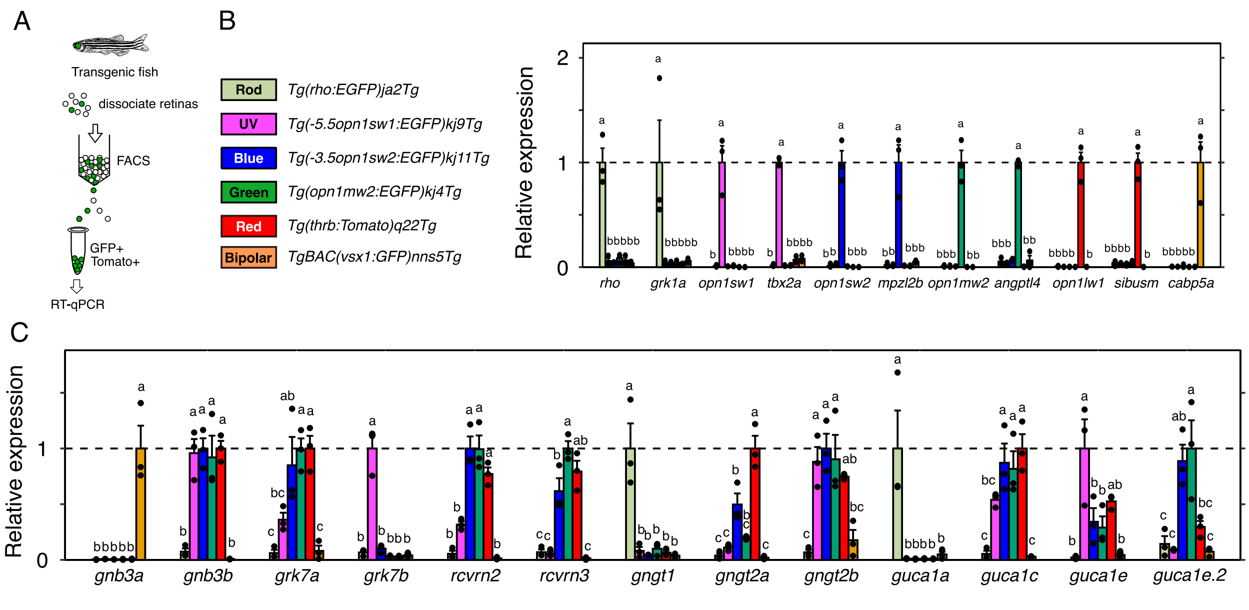

Figure S3. RT-qPCR analysis of differentially expressed genes using cDNA from isolated photoreceptors subtypes and bipolar cells

(**A**) Approach for isolating rods, cone subtypes, and bipolar cells from the indicated transgenic zebrafish lines. GFP- or tdTomato-positive cells were collected from adult fish of each line. **(B-C)** RT-qPCR of cluster-enriched marker genes (**B**) and phototransduction genes (**C**). Data are represented as mean ± SEM (n = 3), while individual values are shown as markers. The transcript levels were normalized to ribosomal protein L13a (*rpl13a*) transcript levels. qPCR results were evaluated by Tukey's honest statistical difference (HSD) test. Distinct letters indicate statistically significant differences (P < 0.05). "sibusm" represents *si:busm1-57f23.1*.

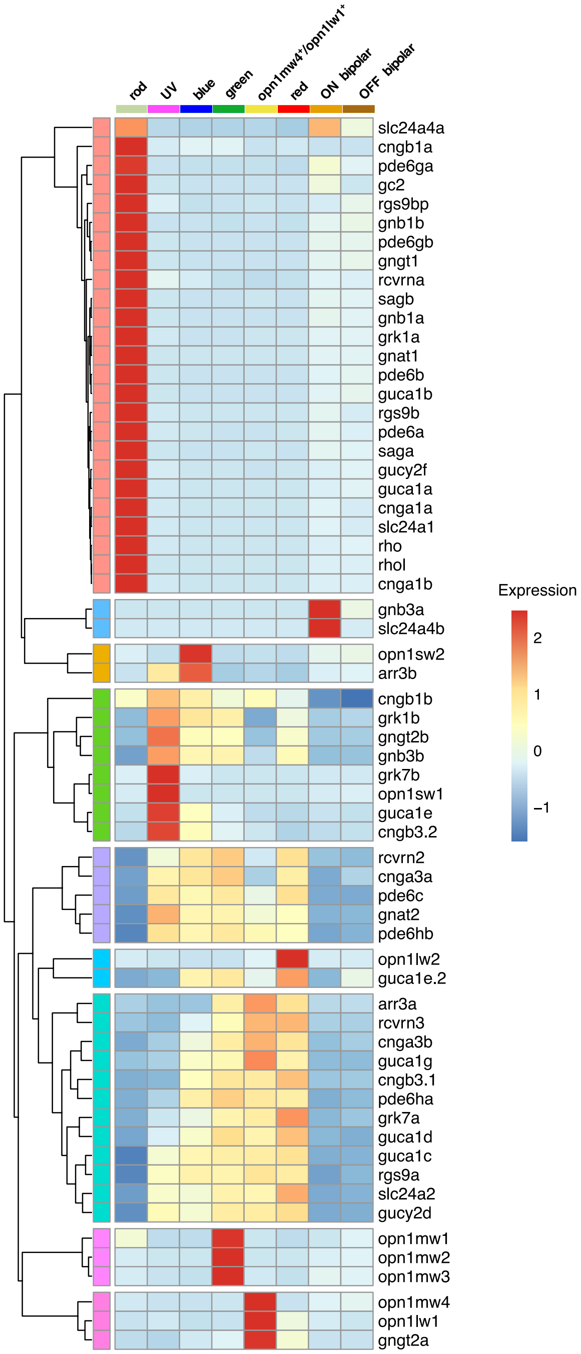

Figure S4. Heatmap showing hierarchically clustered expression patterns of phototransduction genes

Related to Fig. 3C. Heatmap shows average expression levels of genes. Values are row-wise Z-scored gene-expression values. The genes were arranged by hierarchical clustering with the 'complete' agglomeration method using the average gene expression levels. All phototransduction genes except for *guca1e*.2, *guca1g*, *rcvrnb*, and *slc24a4b* were differentially expressed among photoreceptor and bipolar populations.

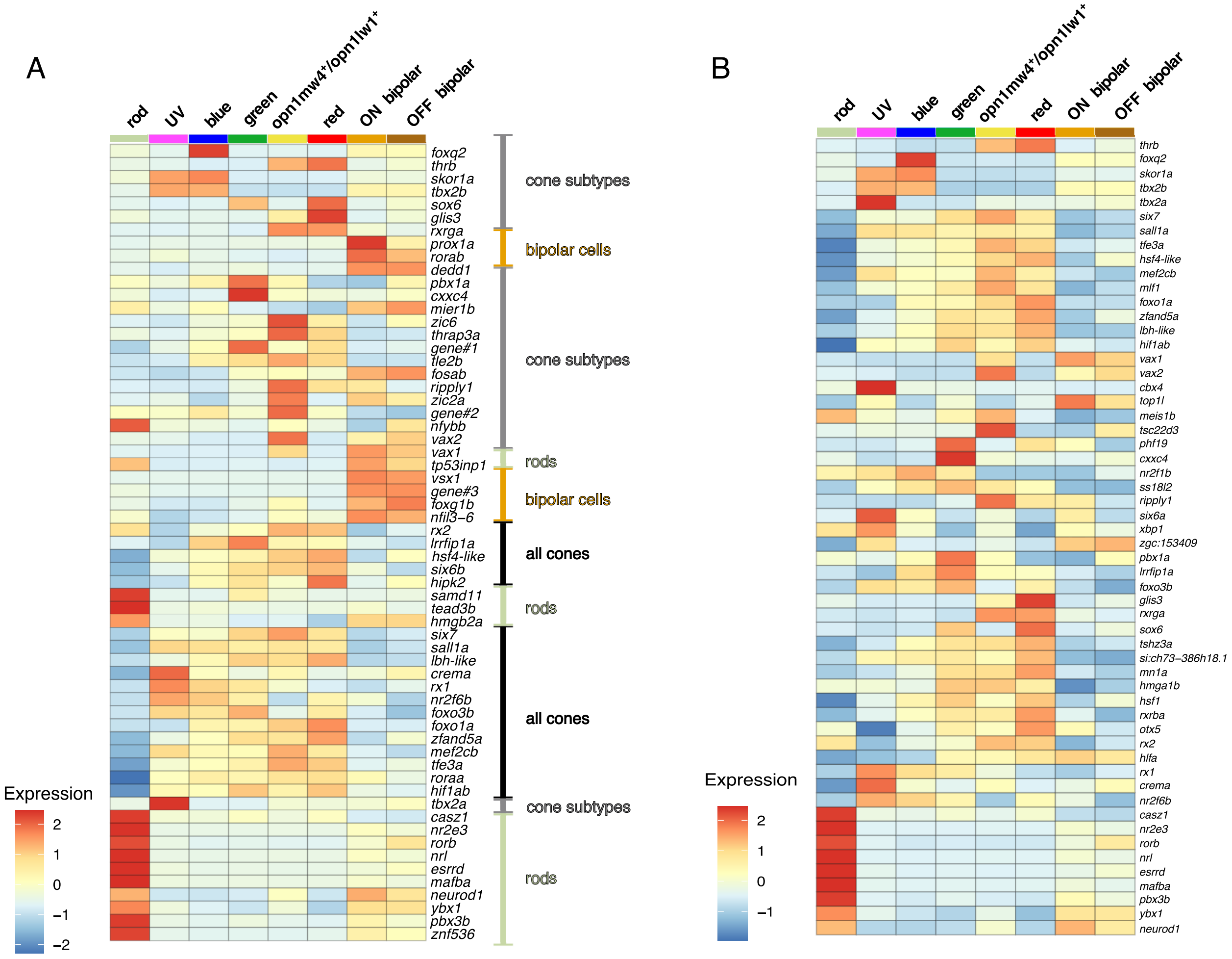

Figure S5. Expression patterns of transcriptional regulators identified by SCENIC analysis

(**A**) Heatmap showing the expression patterns of transcriptional regulators associated with phototransduction genes, presented in the same order as in Fig. 4. Gene#1: zgc:114046; Gene#2: zgc:110269; Gene#3: si:ch211−288g17.3. (**B**) Heatmap showing the expression patterns of transcriptional regulators associated with non-phototransduction genes, presented in the same order as in Fig. S6.

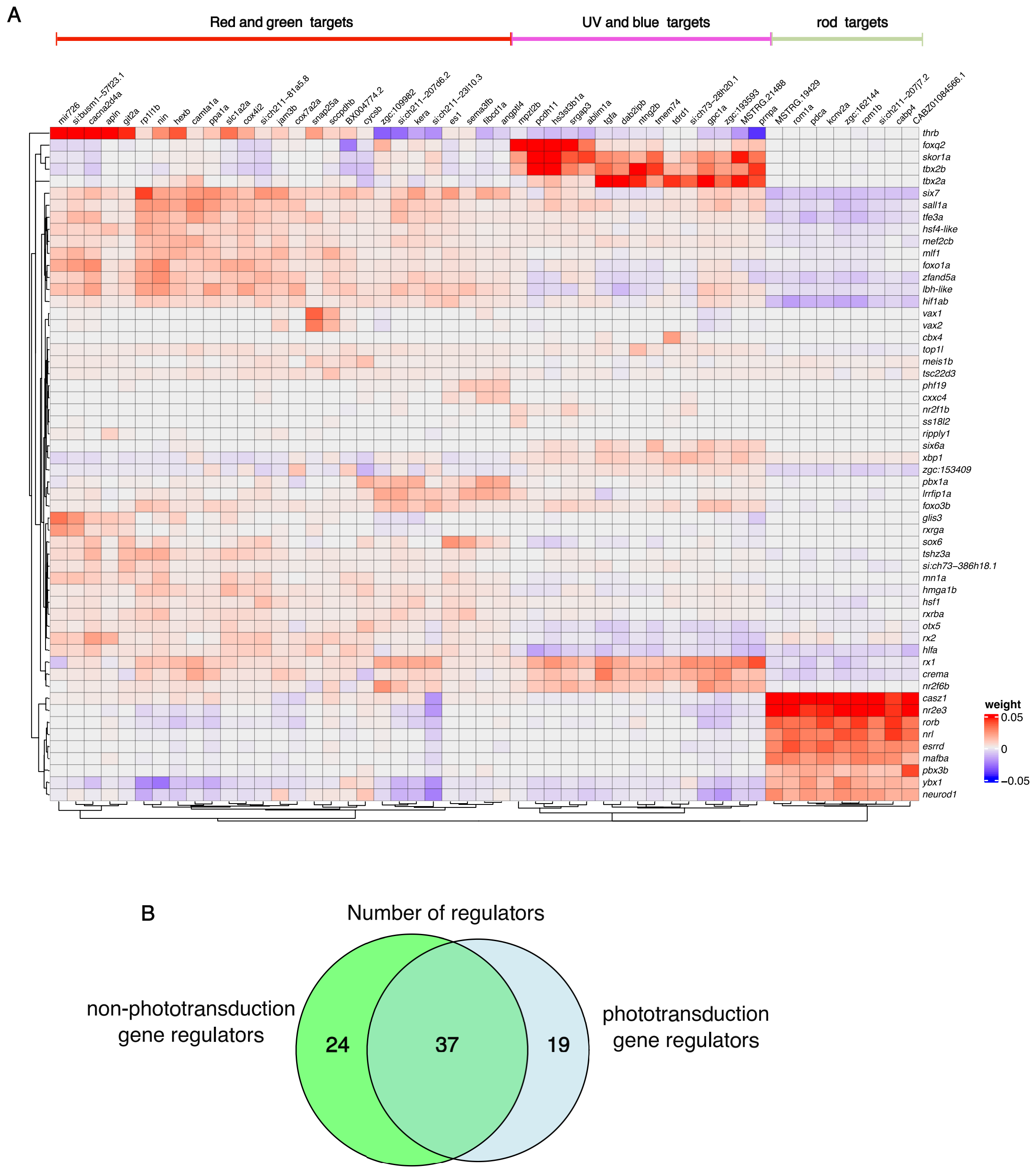
A
Red and green targets
UV and blue targets
rod targets
mir726
si:busm1–57f23.1
cacna2d4a
apln
git2a
rp1l1b
nin
hexb
camta1a
ppa1a
slc1a2a
cox4i2
si:ch211–81a5.8
jam3b
cox7a2a
snap25a
sccpdhb
BX004774.2
cycsb
zgc:109982
si:ch211–207d6.2
kera
si:ch211–23l10.3
es1
sema3fb
fibcd1a
angptl4
mpzl2b
pcdh11
hs3st3b1a
srgap3
ablim1a
tgfa
dab2ipb
ntng2b
tmem74
tdrd1
si:ch73–28h20.1
gpc1a
zgc:193593
MSTRG.21488
prnpa
MSTRG.19429
rom1a
pdca
kcnv2a
zgc:162144
rom1b
si:ch211–207j7.2
cabp4
CABZ01084566.1
thrb
foxq2
skor1a
tbx2b
tbx2a
six7
sall1a
tfe3a
hsf4-like
mef2cb
mlf1
foxo1a
zfand5a
lbh-like
hif1ab
vax1
vax2
cbx4
top1l
meis1b
tsc22d3
phf19
cxxc4
nr2f1b
ss18l2
ripply1
six6a
xbp1
zgc:153409
pbx1a
lrrfip1a
foxo3b
glis3
rxrga
sox6
tshz3a
si:ch73–386h18.1
mn1a
hmga1b
hsf1
rxrba
otx5
rx2
hlfa
rx1
crema
nr2f6b
casz1
nr2e3
rorb
nrl
esrrd
mafba
pbx3b
ybx1
neurod1
weight
0.05
0
–0.05
B
Number of regulators
non-phototransduction
gene regulators
24
37
19
phototransduction
gene regulators

Figure S6. Candidate transcriptional regulators responsible for expression of non-phototransduction genes

Heatmap showing positive (red) and negative (blue) associations between transcriptional regulators (transcription factors and cofactors) and differentially expressed non-phototransduction genes (target genes) calculated by the GENIE3 algorithm in SCENIC. Rows and columns are arranged according to divisive hierarchical clustering (dividing clusters in a top-down manner). The (dis)similarity of observations was calculated using Euclidean distances. Cell type expression patterns of the transcriptional regulators are presented in Fig. S5B. (**B**) Venn diagram showing the number of transcriptional regulators identified by SCENIC analysis using two different sets of target genes: phototransduction gene (Fig. 4) and non-phototransduction genes (Fig. S6A).

Supplementary Data S1. List of genes differentially expressed among adult zebrafish photoreceptors and bipolar cells

"cluster" represents cell cluster, in which the gene is differentially expressed compared to the cells in all the other clusters. "p_val" represents unadjusted p-val. "p_val_adj" represents adjusted p-value, based on bonferroni correction using all genes in the dataset. "avg_log2FC" represents the log fold change of average expression. "pct.1" represents the percentage of cells in the cluster, in which the gene is expressed, while "pct.2" represents the average percentage of cells in all the other clusters, in which the gene is expressed. "seqnames", "strand", "start", "end", and "width" represent gene location in the genome. "original name" is the gene name in our scRNA-seq analysis using the updated transcript annotation (See Methods). "gene" and "Geneid" represents gene name and gene id, respectively, in the annotation file (v4.3.2.gtf) used as an initial guide for the transcript assembly (See Methods). The columns (rod, UV, blue, green, opn1mw4_opn1lw1, red, ON bipolar, and OFF bipolar) represent normalized pseudo-bulk RNA sequencing reads (transcripts per million, TPM).

References

1. Y. Asaoka, H. Mano, D. Kojima, Y. Fukada, Pineal expression-promoting element (PIPE), a cis-acting element, directs pineal-specific gene expression in zebrafish. *Proc. Natl. Acad. Sci. U. S. A.* **99**, 15456–61 (2002).
2. M. Takechi, T. Hamaoka, S. Kawamura, Fluorescence visualization of ultraviolet-sensitive cone photoreceptor development in living zebrafish. *FEBS Lett.* **553**, 90–94 (2003).
3. M. Takechi, S. Seno, S. Kawamura, Identification of cis-acting elements repressing blue opsin expression in zebrafish UV cones and pineal cells. *J. Biol. Chem.* **283**, 31625–32 (2008).
4. T. Tsujimura, A. Chinen, S. Kawamura, Identification of a locus control region for quadruplicated green-sensitive opsin genes in zebrafish. *Proc. Natl. Acad. Sci. U. S. A.* **104**, 12813–8 (2007).
5. S. C. Suzuki, *et al.*, Cone photoreceptor types in zebrafish are generated by symmetric terminal divisions of dedicated precursors. *Proc. Natl. Acad. Sci. U. S. A.* **110**, 15109–14 (2013).
6. Y. Kimura, C. Satou, S. I. Higashijima, V2a and V2b neurons are generated by the final divisions of pair-producing progenitors in the zebrafish spinal cord. *Development* **135**, 3001–3005 (2008).
